# Flowering genes identification, network analysis, and database construction for 837 plants

**DOI:** 10.1093/hr/uhae013

**Published:** 2024-01-10

**Authors:** Tong Wu, Zhuo Liu, Tong Yu, Rong Zhou, Qihang Yang, Rui Cao, Fulei Nie, Xiao Ma, Yun Bai, Xiaoming Song

**Affiliations:** School of Life Sciences/Library, North China University of Science and Technology, Tangshan, Hebei 063210, China; School of Life Sciences/Library, North China University of Science and Technology, Tangshan, Hebei 063210, China; School of Life Sciences/Library, North China University of Science and Technology, Tangshan, Hebei 063210, China; Department of Food Science, Aarhus University, Aarhus 8200, Denmark; School of Life Sciences/Library, North China University of Science and Technology, Tangshan, Hebei 063210, China; School of Life Sciences/Library, North China University of Science and Technology, Tangshan, Hebei 063210, China; School of Life Sciences/Library, North China University of Science and Technology, Tangshan, Hebei 063210, China; School of Life Sciences/Library, North China University of Science and Technology, Tangshan, Hebei 063210, China; College of Horticultural Science & Technology, Hebei Normal University of Science & Technology, Qinhuangdao, Hebei 066600, China; School of Life Sciences/Library, North China University of Science and Technology, Tangshan, Hebei 063210, China; School of Life Sciences/Library, North China University of Science and Technology, Tangshan, Hebei 063210, China

## Abstract

Flowering is one of the most important biological phenomena in the plant kingdom, which not only has important ecological significance, but also has substantial horticultural ornamental value. In this study, we undertook an exhaustive review of the advancements in our understanding of plant flowering genes. We delved into the identification and conducted comparative analyses of flowering genes across virtually all sequenced angiosperm plant genomes. Furthermore, we established an extensive angiosperm flowering atlas, encompassing a staggering 183 720 genes across eight pathways, along with 10 155 ABCDE mode genes, which play a pivotal role in plant flowering regulation. Through the examination of expression patterns, we unveiled the specificities of these flowering genes. An interaction network between flowering genes of the ABCDE model and their corresponding upstream genes offered a blueprint for comprehending their regulatory mechanisms. Moreover, we predicted the miRNA and target genes linked to the flowering processes of each species. To culminate our efforts, we have built a user-friendly web interface, named the Plant Flowering-time Gene Database (PFGD), accessible at http://pfgd.bio2db.com/. We firmly believe that this database will serve as a cornerstone in the global research community, facilitating the in-depth exploration of flowering genes in the plant kingdom. In summation, this pioneering endeavor represents the first comprehensive collection and comparative analysis of flowering genes in plants, offering valuable resources for the study of plant flowering genetics.

## Introduction

**Figure 1:**
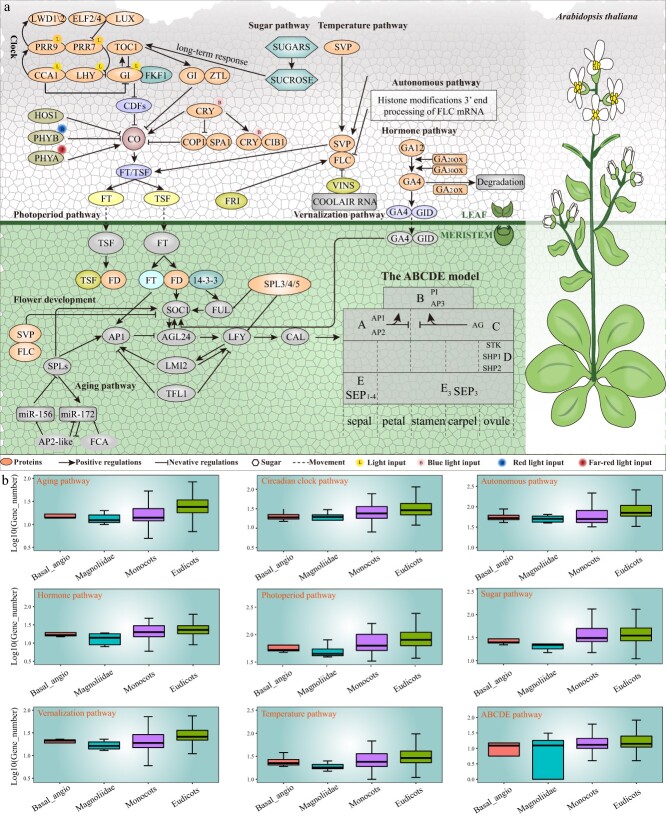
The overview of flowering gene regulatory pathways in plants. **a** The regulatory network by summarizing the interrelationships of key flowering genes in eight pathways and the ABCDE model. **b** The comparative analysis of flowering gene number in different pathways among four classifications of 837 species.

Flowering is the most important biological behavior of angiosperms after a period of vegetative development [1, 2]. Flowering is induced and regulated by a series of factors, which are mainly divided into two categories. One of these is environmental signals, such as temperature, photoperiod, and vernalization [3, 4]. The other one is the endogenous changes of plants, such as hormone level, sugar level, and plant age [5, 6]. Genetic and molecular studies revealed that many genes related to flowering are involved in several regulatory pathways, including photoperiod, vernalization and autonomous, circadian clock, temperature, aging, hormone, and sugar pathways (Fig. 1) [7, 8].

### Photoperiod pathway

*CONSTANS* (*CO*) and *FLOWERING LOCUS T* (*FT*) genes are the two core genes in photoperiod pathway. CYCLING DOF FACTORs (CDFs) are a single zinc finger DOF (DNA binding with One Finger) transcription factor [9]. In the morning, CDFs can bind to the CTTT site of the promoter of *CO* gene and inhibit its transcription [10]. In the afternoon, the accumulation of *CO* reached a peak, and CRYPTOCHROME (CRY) inhibited its activity by binding to the complex of CONSTITUTIVE PHOTOMORPHOGENIC 1 (COP1) and SUPPRESSOR OF PHYA-105 1 (SPA1) [11, 12]. *CO* binds to the promoter of *FT* to activate its expression [13]. FT protein, called florigen, is the key integration point of plant flowering [14]. FT protein can integrate multiple flowering pathway signals and transmit them to downstream factors to regulate plant flowering [15]. Previous studies showed that FT protein is induced in leaves [16]. FT protein is transported to the apical meristem through the vascular bundle system [17]. In meristem, FT, FLOWERING LOCUS D (FD), and 14-3-3 s combine to form the florigen activation complex (FT-FD-14-3-3 complex), which recognize and activate downstream genes, such as *AP1*, and *SUPPRESSOR OF OVEREXPRESSION OF CONSTANS1* (*SOC1*) in *Arabidopsis thaliana* [18]. Both *FT* and *FD* gene mutations can down-regulate or delay the expression of *SOC1* gene [19]. Phytochrome A (phyA), phyB, and cry2 are the vital photoreceptors that regulate flowering [20].

### Vernalization and autonomous pathway

Vernalization is necessary for removing or overcoming the block of flowering, enabling plants to flower in spring or early summer [21]. The vernalization-responsive overwintering plants contain winter annuals, biennials, and perennials [22, 23]. The shoot apical meristem is the important part in response to low temperature [24]. *FLOWERING LOCUS C* (*FLC*) is expressed in most organs of *A. thaliana*. *FLC* is the significant repressor of flowering during vernalization, and it encodes a MADS-box transcription factor [25]. High expression levels of *FLC* gene act as a repressor by repressing the expression of floral integrators genes, such as *FT* and *SOC1* [26, 27]. By inhibiting the expression of *FLC*, vernalization can relieve the inhibition of *FLC* on downstream genes [28, 29]. Activation of *VERNALIZATION INSENSITIVE1* (*VIN1*) can form a dimer with *VIN3* and participate in the methylation and deacetylation of *FLC*, which leads to repression of the expression of *FLC* [30]. *VIN3* is related to histone deacetylation, which can be induced by low temperature to inhibit the expression of *FLC* [31]. In addition, *VIN3* combines with *VERNALIZATION5* (*VRN5*) to form a heterodimer during vernalization, which maintains the histone modification that is necessary for *FLC* silencing [32].

In the absence of external conditions, plants can be regulated by endogenous pathways to make them bloom at an appropriate time. In the autonomous pathway, the transcription level of *FLC* is regulated mainly by changing the structure of chromatin, or by regulating the mRNA content of transcribed *FLC*. The key factors involved in flowering regulation in *A. thaliana* can be divided into mRNA binding protein and chromatin remodeling protein [33]. The mRNA binding protein can bind *FLC* precursor mRNA to regulate the expression of *FLC*. Chromatin remodeling protein inhibits *FLC* expression by epigenetic modification of *FLC* chromatin histone.

### Circadian clock pathway

The circadian clock pathway consists of three negative feedback loops. One is the input pathway through photosynthetic temperature sensors such as phy or cry to receive environmental signals and transmit them to the central oscillator [34]. One is the central oscillator of the circadian clock. The output pathway caused by the change of rhythm regulated by the circadian clock [34]. In circadian clock pathway, *CIRCADIAN CLOCK ASSOCIATED 1* (*CCA1*) and *LATE ELONGATED HYPOCOTYL* (*LHY*) are expressed in the early morning [35]. *CCA1* and *LHY* can form allodiploids and play a synergistic role in the biological clock pathway [35]. They inhibit the transcription of *FLAVIN-BINDING KELCH REPEAT F-BOX 1* (*FKF1*) and *GIGANTEA* (*GI*) genes [36]. *GI* is a nocturnal clock gene, which inhibits the transcription of *CCA1* and *LHY* [36]. *FKF1* and *GI* interact with each other in a blue-light-dependent manner, and they can degrade *CDFs* to promote flowering in *A. thaliana* [37]. The degradation of *CDFs* makes *CO* accumulate in the afternoon [38]. In addition, *GI* can also bind to *ZEITLUPE* (*ZTL*), which indirectly inhibits the binding of *GI* to *FKF1* [39].

The second feedback loop is mediated by *PSEUDO-RESPONSE REGULATOR7* (*PRR7*) and *PRR9*, and the transcription of these genes is promoted by *LHY* and *CCA1* [42]. The accumulation of PRR7 and PRR9 leads to down-regulation of *LHY* and *CCA1* transcription [19, 40]. ELF3/ELF4/LUX forms a nocturnal complex that inhibits transcription. ELF3/ELF4/LUX inhibits the transcription of *PRR9*/*PRR7* through the combination of LUX and the promoter of *PRR9*/*PRR7*, which leads to the disappearance of the inhibitory effect of *PRR9*/*PRR7* on *CCA1*/*LHY* transcription [41, 42]. The central feedback loop, morning feedback loop and night feedback loop constitute the feedback loop of the circadian clock pathway in *A. thaliana*.

### Temperature pathway

Temperature is the most important environmental signal to regulate plant growth and development, especially in the key stage of reproduction [43]. A suitable high temperature (27°C) can make *A. thaliana* blossom earlier [44]. Low temperature (16°C) inhibited plant flowering. The selective splicing of *FLM* transcripts is temperature dependent [45]. *FLM* is mainly *FLM*β at low temperature and *FLM*δ at high temperature [46]. Both of them can interact with *SVP* competitively, which results in the difference of the abundance of SVP-FLM-β repressor at different temperatures [47]. This leads to the difference of flowering time in the end. Meanwhile, SVP degraded at high temperature, resulting in a decrease in the abundance of FLM-SVP-β inhibitors and further promoting plant flowering [48]. MiR156 and miR172 are also involved in the process of environmental temperature regulation of flowering [49]. Low temperature could induce the product of miR156, whose target genes are in *SPL3* [49]. Therefore, the mRNA level of *SPL3* is decreased by the increasing expression of miR156 at low temperature [50]. *SPL3* can directly activate the expression of *FT*, thus regulating the temperature-dependent flowering pathway [50].

### Aging pathway

MiR156 and miR172 are important members of the age pathway. The concentrations of SQUAMOSA PROMOTER BINDING LIKE (SPL) transcription factors increase as the plant ages, which are negatively regulated by miR 156 [51, 52]. The expression level of miR156 in young plants was high and decreased with age [53]. In this way, the *SPL* family genes, which are the target genes of miR156, will gradually increase with age [53]. SPL3, SPL4, and SPL5 can directly activate flowering genes such as *FT*, *SOC1*, and *AP1* to induce flowering [53]. The expression pattern of miR172 is opposite to that of miR156, and the expression of miR172 increases with age [54, 55]. SPL9 can promote flowering by promoting the expression of miR172 and inhibiting the expression of *AP2-like* flowering inhibitors [56].

### Hormones pathway

Gibberellic acids (GAs) are required for almost all major plant developmental processes, such as seed germination, stem elongation, leaf expansion, and flowering by promoting cell division and elongation [57, 58]. It has been studied that GA signaling regulates flowering in *A. thaliana* [59]. Under long-day conditions, GA in leaf and stem tip can promote flowering, while it leads to a non-flowering phenotype under short-day conditions [59]. *DELLA* degradation is necessary to activate flowering [60]. As a central inhibitor of GA signaling pathway, *DELLA* has been shown to interact with many transcription factors to regulate flowering [61]. In addition, *DELLA* can bind *CO* through its CCT (CONSTANS, CONSTANS-LIKE, TOC1) domain and resist *CO* from binding to *FT* promoter [62].

Cytokinins is a plant hormone that regulates the growth and development of plant cells. Early studies have shown that cytokinin or gibberellin can induce flower bud formation by activating the expression of *SaMADS*, which belongs to *MADS-box* gene [63]. Auxin can promote the growth of floral organs and the development of pistils in angiosperms [64]. The polar transportation of auxin is necessary for flower bud formation in *A. thaliana* [65]. Auxin response factor *(ARF3*) may affect the establishment of the boundary between ovules in pistil by responding to the gradient of auxin concentration [66].

### Sugar pathway

NO participates in many important physiological processes, and the source of NO in plants is abundant [67]. In the photoperiod pathway, *CO*, as the downstream element, plays an important role in connecting the circadian rhythm with the formation of floral meristem [68]. When the central regulatory components of circadian clock, *TOC1* and *CCA1*, are normally expressed, endogenous NO can inhibit the signal transmission from circadian clock to photoperiodic pathway, thus reducing the expression of *GI* and its downstream *CO* gene [69]. The low expression of *CO* down-regulated the expression of *LFY* gene, prolonged the vegetative growth, increased the number of rosette leaves, and delayed flowering [70]. Sucrose is an important signal molecule that affects flowering in plant [71]. Interestingly, sucrose supply reduced the level of S-nitrosation of GI and CO proteins, which counteracts the flowering delay induced by NO [68]. Previous studies have revealed that S element reverses the phenotype of late flower caused by NO and may be related to its antagonistic regulation of photoperiod gene expression [67].

### ABCDE model

The ABCDE model effectively explains the molecular mechanism of floral organ development and variation in *A. thaliana* [72,73]. This model assumes that regulatory genes work in combination to give organ identity [74]. Among ABCDE model, the protein complex develops sepals as the ground-state floral organs in the first floral whorl when A- is combined with E- class [75]. When A-, B-, and E-class are combined, the protein complex specifies petals in the second whorl [76]. When B-, C-, and E-class are combined, the protein complex specifies stamens in the third whorl [76]. When C- is combined with E- class, the protein complex specifies carpels in the fourth whorl [77]. *APETALA1* (*AP1*) and *AP2* belong to A-class; *AP3* and *PISTILLATA* (*PI*) belong to B-class; *AGAMOUS* (*AG*) belongs to C-class; *SEEDSTICK* (*STK*), *SHATTERPROOF1* (*SHP1*), and *SHP2* belong to D-class; *SEPALLATA1* (*SEP1*), *SEP2*, *SEP3*, and *SEP4* belong to E-class [77, 78]. Except for *AP2*, all ABCDE model genes contain a highly conserved DNA sequence of about 180 bp, called MADS-Box [79]. The diversification of MADS-box family genes in the evolutionary process has greatly contributed to the extensive changes of flower patterns in plants [80–82].

## Results

### Flowering gene identification and database construction

Based on the isolation of loss-of-function mutations or analysis of transgenic plants, we collected 327 flowering genes in the model plant *A. thaliana* from 1548 publications (Table S1, see online supplementary material). In addition, many plant genomes have been sequenced in recent years, providing rich resources for research on flowering genes. After a serial genome data collection and filtering, 837 species with high-quality gene annotation were used in this study, including four basal angiosperms, 11 magnoliidaes, 160 monocots, 662 eudicots (Table S2, see online supplementary material). We have sorted out the whole genome duplication (WGD) events of these species by class (Fig. S1, see online supplementary material). Moreover, it has been reported that there are a lot of homologous genes of *A. thaliana* regulating flowering in other angiosperm species [83]. Therefore, we used Blast (Identity >70%) to make homologous comparisons between the *A. thaliana* flowering genes and angiosperm genomes.

In this study, we identified many important flowering genes, including 80 810 genes in the photoperiod pathway, 34 373 genes in the sugar pathway, 30 396 genes in the temperature pathway, 27 451 genes in the vernalization pathway, 22 839 genes in the aging pathway, 73 286 genes in the autonomous pathway, 29 511 genes in the circadian clock pathway, 24 707 genes in the hormone pathway, and 10 155 ABCDE model genes (Fig. 1b; Table S3, see online supplementary material). Furthermore, we predicted microRNAs (miRNAs) target genes for these flowering genes and generated the regulation network diagrams for further functional genomics study. In addition, visitors can also submit the flowering genes which they want to predict so that they can conduct further analysis and we conducted flowering genes expression pattern analyses in representative species using RNA-seq data and other public expression datasets to explore their expression pattern.

Finally, we established a data sharing and comparative analysis platform for plant flowering genes called the Plant Flowering-time Gene Database (PFGD, http://pfgd.bio2db.com/) based on all of the results above. We believe it can provide convenient retrieval and download services for more researchers related to flowering genes.

### Network construction of flowering genes

We identified the upstream regulatory sequences of flowering genes and ABCDE genes using PlantPAN 4.0 (http://plantpan.itps.ncku.edu.tw/plantpan4/index.html). Then, we made these sequences accessible in the ‘Browse-Pathway-Upstream genes’ section of our PFGD website for users to view and download. Here, we selected the 12 ABCDE model genes in *A. thaliana* and showed the relationship between them and their upstream regulated genes (Table S4, see online supplementary material). These datasets and main results were deposited in our database, which provided rich resources for the study of floral organ development and the evolution of genes from the ABCDE model in plants.

**Figure 2:**
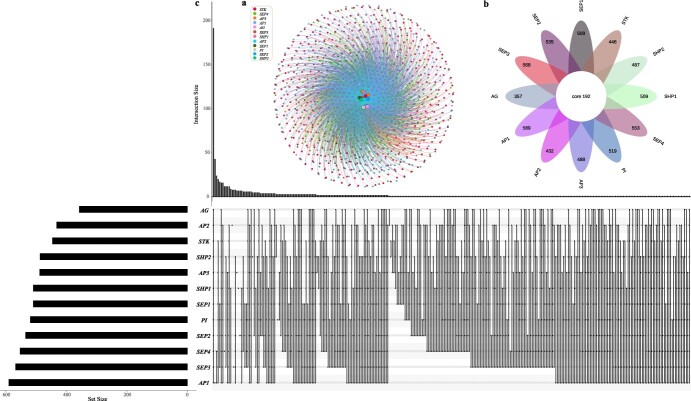
The interaction network between the flowering genes (ABCDE model) and the corresponding upstream genes in *Arabidopsis thaliana.***a** The network between the flowering genes and the corresponding upstream genes. **b** The number of the common and the specific upstream genes in each flowering gene. **c** The display of all original quantity and intersection of each group.

A total of 901 upstream genes of the 12 ABCDE model genes In *A. thaliana* were detected (Table S5, see online supplementary material). Then, we constructed the interaction networks according to the relationship of ABCDE model genes and their corresponding upstream genes (Fig. 2a). This regulatory relationship provides the resources for further verification of the function of flowering genes. Then, the Venn diagrams were used to show the common or specific relationship of these upstream genes for each ABCDE model gene (Fig. 2b and c, , see online supplementary materialTable S6). The results showed that there were 192 upstream regulatory genes that are shared by all 12 ABCDE model genes (Fig. 2b). These 192 core upstream regulatory genes may play an important role in regulating flowering. Among them, *AP1* (class A) had the most upstream regulatory genes (589 genes), followed by SEP3 (568 genes, class E) and SEP4 (553 genes, class E), while *AG* (class C) had the least (357 genes). In addition, we analysed and showed the gene number of different groups and the specific number of all the intersections and situations between them (Fig. 2c). Then, we identified the families of the upstream genes and performed the enrichment analysis based on Pfam annotation (Table S7, see online supplementary material). A total of 12 items were significantly enriched, of which the WRKY gene family was the most significant (*q*-value <2.38 × 10^−79^). This result was consistent with the previous reports that the genes of the WRKY gene family promote flowering [84, 85]. Our findings point out a new direction for studying the functions of *WRKY* genes involved in flowering and lay a foundation for studying the relationship between ABCDE model genes and *WRKY* family genes or other enriched functional terms detected in this study.

### Exploring flowering-related *cis*-regulatory elements and the regulatory relationships in three-dimensional genomic data

**Figure 3:**
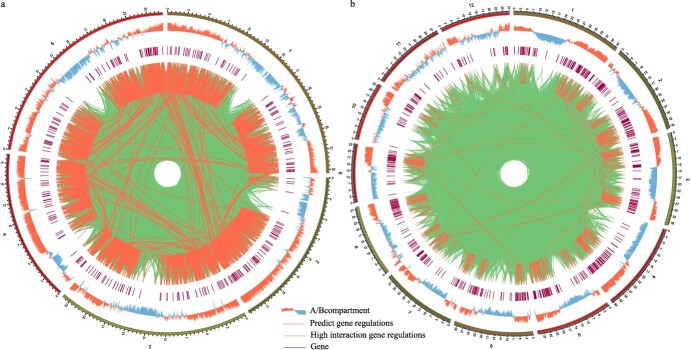
Flowering gene related *cis*-regulatory elements and the regulatory relationships in three-dimensional genomic data. **a** Flowering gene related *cis*-regulatory elements and the regulatory relationships in three-dimensional genomic data of *Arabidopsis thaliana.***b** Flowering gene related *cis*-regulatory elements and the regulatory relationships in three-dimensional genomic data of * Oryza sativa*.

The spatial arrangement of DNA can influence interactions between regulatory factors and flowering genes. We collected Hi-C data for both *A. thaliana* and *O. sativa*, conducting a comprehensive three-dimensional genome analysis. Our results revealed a significant concentration of flowering genes within region A, corresponding to areas characterized by more accessible chromatin structures. Using *A. thaliana* as an example, we created a *A. thaliana* chromatin interaction matrix with a 20-kb resolution. Within this matrix, we identified a total of 110 123 potential interaction sites between transcription factors and flowering genes, resulting in 24 526 pairs of interaction intervals. We quantified the interaction strength within these intervals, with the majority registering a strength of 1. As a result, we focused on intervals with interaction strengths greater than 5, speculating there are stronger interactions than typical regions. This led us to select 2356 interval pairs, constituting 9.6% of all interval pairs. Subsequently, we identified 1929 pairs of regulatory factors and flowering genes based on the positional information from these highly interactive intervals (Fig. 3a; Table S8, see online supplementary material). We also conducted a similar analysis in *O. sativa*, uncovering 517 high-strength interaction interval pairs, accounting for 4.6% of all interval pairs, which included 569 pairs of regulatory factors and flowering genes (Fig. 3b; Table S9, see online supplementary material). We hypothesized that these regulatory relationships may play a significant role in modulating the expression of flowering genes within a three-dimensional spatial context.

### Exploring the expression pattern of ABCDE model genes in representative species

We selected the transcriptome data of six representative species to explore the expression pattern, including four eudicots (*A. thaliana*, *Vitis vinifera*, *P. equestris*, and *Cucumis melo*), 1 monocot (*O. sativa*), and 1 basal angiosperm (*Amborella trichopoda*) (Tables S10–S15, see online supplementary material).

We have explored the expression pattern of ABCDE model genes in these species, and the heatmaps of transcriptome data have shown their action site and expression level in different species (Figs S2–S7, see online supplementary material). We found that flowering genes had higher expression in floral organs and seeds in *A. thaliana*, *P. equestris*, *V. vinifera*, and *O. sativa*. Seed germination and flowering are two critical developmental transitions in plants. Previous studies have found that the genes of the autonomous pathway regulate seed germination in *A. thaliana* [86]. In addition, *FLC* has been confirmed to influence germination in *H. vulgare* and *Triticum aestivum* [87]. Furthermore, ABCDE model genes of *A. trichopoda* have a wide expression profile in various organs, indicating that ABCDE model genes in early angiosperms may be widely expressed, but strictly expressed in monocots and dicots. The expression of ABCDE model genes in *A. trichopoda* may also explain that sepals and petals do not differentiate in a real sense, which is similar with the ancestors of angiosperm flowers [88]. Therefore, this study can lay a foundation for a better analysis of expression patterns for flowering genes, which provides rich resources for understanding their flowering mechanism and evolution footprint.

### Identification of miRNAs and target genes

To better study the regulation mechanism of miRNAs related with flowering, we have identified the target genes of flowering-related miRNAs in all examined species (Table S16, see online supplementary material). We collected 14 main miRNAs related to flowering from *A. thaliana*. Based on these miRNAs, we predicted their target genes in other species. A total of 5700 target genes was detected in 837 species. There were the most target genes in *Zingiber officinale* [47], followed by *Camelina sativa* [41] and *Brassica napus* [40]. Then, we generated the regulation network between miRNAs and their target genes, which can be found in our PFGD database. We found several miRNAs can have multiple target genes, such as miR156 and miR172. Similarly, miR156 and miR172 can also regulate the same target gene. The miRNAs are closely related to the evolution of their target genes, and the study of their relationship is helpful to further understand their mechanism and function in flowering. All of these results were deposited in our PFGD database.

### Plant Flowering-time Gene Database (PFGD) construction

**Figure 4:**
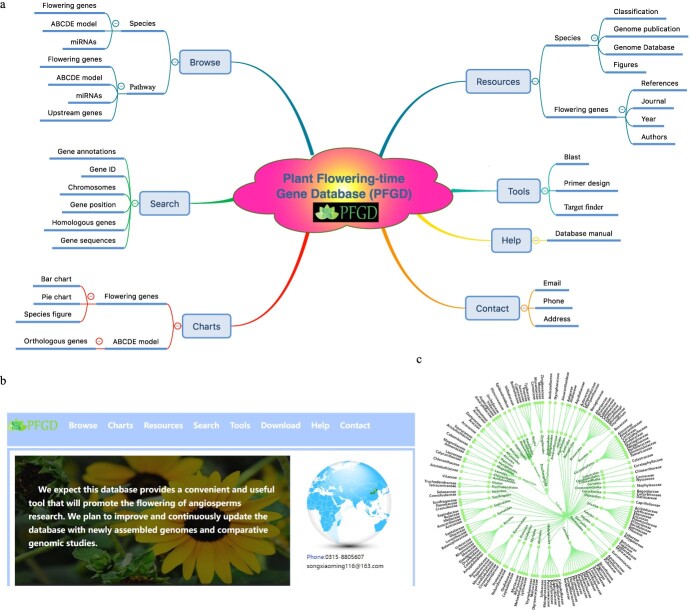
Overview of the PFGD database. **a** The architecture of the Plant Flowering-time Gene Database (PFGD). **b** The home page of the PFGD. **c** The classification of 837 species.

**Figure 5:**
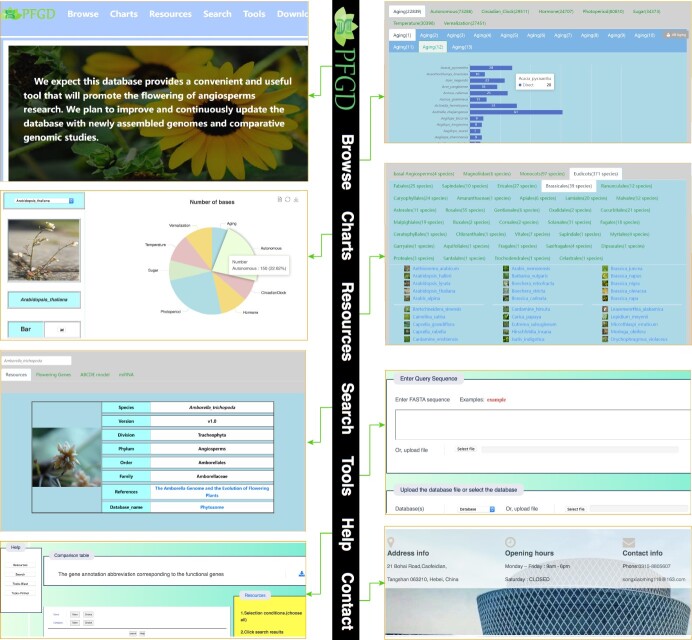
Overview of the PFGD with its main interfaces and internal features, including home, browse, charts, resources, search, tools, help, and contact interfaces.

By summarizing and collating the above-mentioned data, the comprehensive flowering gene database was constructed, which included 11 modules: aging, autonomous, circadian clock, hormone, photoperiod, sugar, temperature, vernalization, ABCDE model, miRNA target genes, and expression pattern. We built the comparative and functional genomic database with a user-friendly web interface (PFGD, http://pfgd.bio2db.com/) based on the 837 released angiosperms’ genomes (Fig. 4). The PFGD database contains the detailed information of many important flowering genes. Moreover, PFGD also integrated the miRNA target genes related to the flowering in 837 species. Therefore, this is the first large-scale analysis of flowering genes using the angiosperms genomic data. All the genes from different flowering pathways and bioinformatics results can be easily searched or downloaded from the PFGD database. The Blast and Primer design are provided to help users perform comparative and functional genomic analyses of flowering genes in more species. The TargetFinder is provided to predict the relationship between target genes and miRNA in each species. Here, we present an overview of the interfaces of the PFGD database, including the Browse, Charts, Resources, Search, Tools, Help, and Contact interfaces, to help users easily use our database (Fig. 5).

### Search

Users could search the flowering genes of eight pathways in each species. The users also can obtain the related genes of all species at one time by selecting ‘All’ in the drop-down frame. Search results contain rich information, including gene name, position (chromosome, start, end), homologous with *A. thaliana* genes (identity, E-value, score), annotation, CDS, and protein sequences. Most importantly, we provide a download function for search results. Users can further conduct subsequent cross-species comparative analysis on the results.

### Browse

We provide two modes, ‘Species’ and ‘Pathway’, for users to browse the flowering gene data.

In ‘Species’ mode, we have classified species, and users can easily locate their own species according to the classification of the queried species. Then users can query all eight pathways of the species for flowering genes and ABCDE model genes. At the same time, we also provide a download function for query results, so that users can easily download relevant data for further research. Using 14 miRNAs related to flowering in Arabidopsis, we identified the miRNA target genes of each species. Meanwhile, the results are displayed, including miRNAs, target genes, score, range, strand, target, and query sequences. Moreover, we also provided the three-dimensional genomic data of flowering genes in *A. thaliana* and *O. sativa*.

In ‘Pathway’ mode, users can view the related genes of all species in a pathway or gene family of ABCDE model at one time, and we display the number of genes in each species through a bar chart. Moreover, we identified transcription factors (TFs) that regulate the flowering genes of each ABCDE model genes in Arabidopsis. At the same time, the identification results are displayed on the website, including the TF name, TF family, position, and binding sequence.

### Charts

Through the chart interface, users can easily view the number of flowering genes of each pathway in each species through a pie chart or bar chart. Meanwhile, users can also view the number of ABCDE model genes in each species.

### Tools

We provided three popular tools, Blast, Primer Design, and TargetFinder, to help users to conduct the flowering gene analysis. The Blast tool was used to help users perform CDS or protein sequence alignment. We constructed a user-friendly interface and built the Blast databases using the flowering genes of each pathway from 837 species. All users easily conducted sequence alignment by copying the sequences to the frame or uploading sequence file in Fasta format. We also developed a Primer Design tool to help users to design the primers for the flowering genes or other assigned genes. In addition, we provided visitors with a miRNA target so that they can analyse the relationship between miRNAs and flowering-related genes.

### Resources

In the Resources section, we provided the Latin name, taxonomy, genomic information, published articles, and data download links of the species used in this study. In addition, we also provide details of Arabidopsis flowering genes, including gene name, pathway, references, journal, publication year, and authors. Users can directly download this information for their research.

### Help and contact

In the Help section, a detailed manual was provided for users to know how to use each interface of our database. In addition, we also provided the address, email, and phone number for users to contact us for any related question.

## Discussion

In plants, flowering is the most biological behavior of angiosperms, which give rise to successful sexual reproduction and seed development, but there are few studies that have described the comprehensive regulatory relationships of the flowering genes in large-scale angiosperms. The Flowering Interactive Database website (http://www.phytosystems.ulg.ac.be/florid/) exclusively presents data on flowering genes in Arabidopsis, providing an incomplete resource for the comprehensive study of flowering genes within angiosperms. Additionally, this website ceased its updates in 2016. As a result, there is a critical demand for the development of a new, all-encompassing database dedicated to flowering genes in angiosperms. Such a database would serve as a robust cornerstone for in-depth research into the realm of flowering genes.

Based on the results of previous studies, we collected all genes related to flowering. This approach significantly enhances the authenticity and reliability of the data. Furthermore, we categorized the integrated flowering genes into 19 gene families based on their domains. Interestingly, we observed that the flowering-related *LEAFY* (*LFY*) gene family comprises 118 same gene sequences in the Hormone, Sugar, Photoperiod, Vernalization, Temperature, and Aging pathways, spanning 86 species. This suggests that the *LFY* family primarily functions in the six flowering pathways, reinforcing the accuracy of functional gene identification. Additionally, all genes within the HLH domain were found to belong to the *bHLH* family, further confirming the precision of domain identification.

Here, we have drawn an overview of their regulatory relationships by summarizing the interrelationships of key genes in these flowering gene pathways. More than 344 068 flowering genes from eight pathways and the ABCDE model were identified in the genome of 837 plants. This is the first time there has been exploration of flowering genes in such a large-scale species. In addition, we have built a database platform for users to browse and download flowering genes. We believe this database will lay a solid foundation for further exploration of flowering genes.

Furthermore, we also explored the miRNAs and their target genes related to the flowering in these plants. Previous studies indicate that microRNA156 targets gene *SQUAMOSA PROMOTER BINDING PROTEIN-LIKE* (*SPL*), which acts downstream of *FT*/*FD* genes [89]. High levels of miR156 in young plants prevent flowering [90]. MiR172 is required for regulating organ identity and flowering time by targeting *AP2* gene [91]*.* MiR159 plays a significant role in normal anther development by targeting *MYBs* and controlling their expression [92]. MiR169, whose target gene is *ATNF-YA2*, is necessary for flowering in *A. thaliana*, *Zea mays*, and *Glycine max* under abiotic stress [93]. In this study, we obtained 9294 target genes of 14 miRNAs involved in flowering. These reports revealed that miRNA-mediated genes are essential for plant flower development. The genes provide abundant data resources for the study of miRNA mediated flowering in plant.

Moreover, we established a data sharing and comparative analysis platform for plant flowering genes called Plant Flowering-time Gene Database (PFGD, http://pfgd.bio2db.com/) based on the now released angiosperms’ genomes. We also performed identification, comparative analysis, and database construction of the flowering genes. We hope it can provide a convenient platform for researchers related to flowering genes in plants.

## Conclusion

This is the first large-scale collection of angiosperms flowering data, and it provides a wealth of resources for flowering research in plants. The phylogenetic trees of the orthologous genes belonging to the ABCDE model from all species were constructed for each gene family, and their conserved motifs were also analysed. Furthermore, we analysed the interaction network and expression level of the flowering genes in the different representative species. The interaction network between the flowering genes (ABCDE model) and the corresponding upstream genes provided the blueprint for their regulatory pathways. The expression pattern in different organs of the genes showed their functional divergence.

Finally, we constructed PFGD by integrating these data, which provides rich resources for researchers of flowering genes in plants. Moreover, we have drawn an overview of their regulatory relationships by summarizing the interrelationships of key genes in these pathways. In conclusion, this database will facilitate research of flowering genes studies in plants. Users can easily retrieve and download the target functional genes for research. In the future, we will continuously improve and update the flowering genes of newly assembled genome species in our database. We expect the database to become a key resource for the study of flowering genes for all related researchers in the world.

## Materials and methods

### Identification of genes in flowering regulatory pathways and ABCDE model

Here, the candidate genes obtained with different parameters were compared with the previous literature, and after a series of parameter corrections, the final parameters of Blastp used in this study were *E*-value <1 × 10^−5^ and Identity >70% [94,95]. The 12 ABCDE model genes have been collected in *A. thaliana* (Table S4, see online supplementary material). The homologous flowering regulatory genes between *A. thaliana* and all examined species were identified using Blastp [96]. The retrieved candidates were further verified by the SMART and Conserved Domain Database (CDD) [97, 98].

### Interaction network and enrichment analysis of genes in flowering genes

MEME (v5.3.3) was used to search conserved motifs in the orthologous genes with the motif number set as 10 [99]. The upstream genes of the flowering model genes were obtained from PlantPAN 4.0 (http://plantpan.itps.ncku.edu.tw/index.html) [100]. The interaction networks of those genes were constructed using Gephi software (https://gephi.org) [101]. A Venn diagram (http://bioinformatics.psb.ugent.be/webtools/Venn/) and R program were used to show their overlapping relationship [102]. Then, the Pfam (version 35.0) was used to identify the families of those genes [103]. Furthermore, the script package of Python was used to perform the enrichment analysis based on the result of Pfam [104–106]. The *P*-values obtained by significance analysis were further corrected using the Bonferroni method of the R program [105]. The corrected *P*-value (*q*-value) <0.05 and fold-change >2 was used to define significant enrichment terms.

### Flowering-related *cis*-regulatory elements and the regulatory relationships in three-dimensional genomic regulatory data

Hi-C data underwent preprocessing using the HiC-pro pipeline [107, 108]. The resulting output files, containing only unique and valid read pairs, were utilized for subsequent analysis. We utilized HiCExplorer to generate contact matrices (https://github.com/deeptools/HiCExplorer/releases). The genomes were partitioned into unequal-sized bins based on the genomic positions of restriction sites, and a matrix was constructed using these bins as both rows and columns. Mapped reads were then processed to count the frequency of connections between any two bins through Hi-C read pairs. Chromosomal compartments were identified through principal component analysis (PCA) on 20-Kb resolution contact maps. A distance matrix was generated for each chromosome, and correlations between the contact profiles from different regions were calculated. Subsequently, the first principal component value was derived from the correlation matrix. Using the reference genome annotation file, active regions were assigned. We used the Python program to extract and screen the interaction matrix to map the information of regulatory factors and flowering gene loci into the matrix.

### Expression datasets collection for ABCDE model gene analysis

The expression data of *A. thaliana* was obtained from the Arabidopsis electronic fluorescent pictographic (eFP) Browser (http://www.bar.utoronto.ca/efp/cgi-bin/efpWeb.cgi), and the data of *V. vinifera* was obtained from the Grape eFP Browser (http://bar.utoronto.ca/efp_grape/cgi-bin/efpWeb.cgi) [109]. The data of *C. melo* was obtained from https://melonet-db.dna.affrc.go.jp/ap/top [110]. The data of *O. sativa* was downloaded from Rice eFP Browser (http://bar.utoronto.ca/efp_rice/cgi-bin/efpWeb.cgi) [109]. The transcriptome data of *P. equestris* were downloaded from orchidbase (http://orchidbase.itps.ncku.edu.tw/est/Phalaenopsis_2019.aspx) [111]. The data of *A. trichopoda* was obtained from the related article under specific conditions [112].

### miRNAs and target genes prediction

In *A. thaliana*, a total of 14 main miRNAs related to flowering were collected from publication and TAIR (https://www.arabidopsis.org/index.jsp) database. TargetFinder was used to predict the relationship of microRNA and its target genes by target penalty strategy [113]. Then, we generated their regulation network diagrams by Python scripts.

### Database construction

The PFGD database was constructed with the MySQL database management and Django framework according to the previous reports [114–116]. Several programming languages, including JavaScript, Python, HTML and CSS were used for constructing the PFGD database. The interactive Web interfaces help users to conveniently search the PFGD to obtain the related information according to the previous reports [117–119]. The charts were created using Echarts, which was an open-source visualization tool (https://echarts.apache.org/).

## Acknowledgements

This work was supported by the National Natural Science Foundation of China (32172583), Natural Science Foundation for Distinguished Young Scholars of Hebei Province (C2022209010), Natural Science Foundation of Hebei (C2021209005), and Key Laboratory of Nucleic Research, Tangshan (2022TS003b).

## Author contributions

X.S. conceived the project and was responsible for the project initiation. X.S., T.W., and X.M. supervised and managed the project and research. The data collection and bioinformatic analyses were led by X.S., T.W., Q.Y., X.M., Y.B., and R.C. The database construction was led by X.S., Z.L., T.Y., F.N., and X.M. The manuscript was organized, written and revised by X.S., T.W., X.M., Y.B., and R.Z. All authors read and approved the manuscript.

## Data availability

All materials and related datasets in this study are available in our PFGD database (http://pfgd.bio2db.com).

## Conflict of interest statement

The authors declare no competing interests.

## Supplementary data

Supplementary data is available at *Horticulture Research* online.

## Supplementary Material

Web_Material_uhae013
