# Supplementary material for: Flowering genes identification, network analysis, and database construction for 837 plants
Source: Hortic Res. 2024 Jan 10;11(4):uhae013. doi: 10.1093/hr/uhae013 (PMC10995624; doi:10.1093/hr/uhae013)
Supplement: Web_Material_uhae013 [file web_material_uhae013.zip › Supplementary Figures 1-7.pdf]

Supplementary Figures 1-7

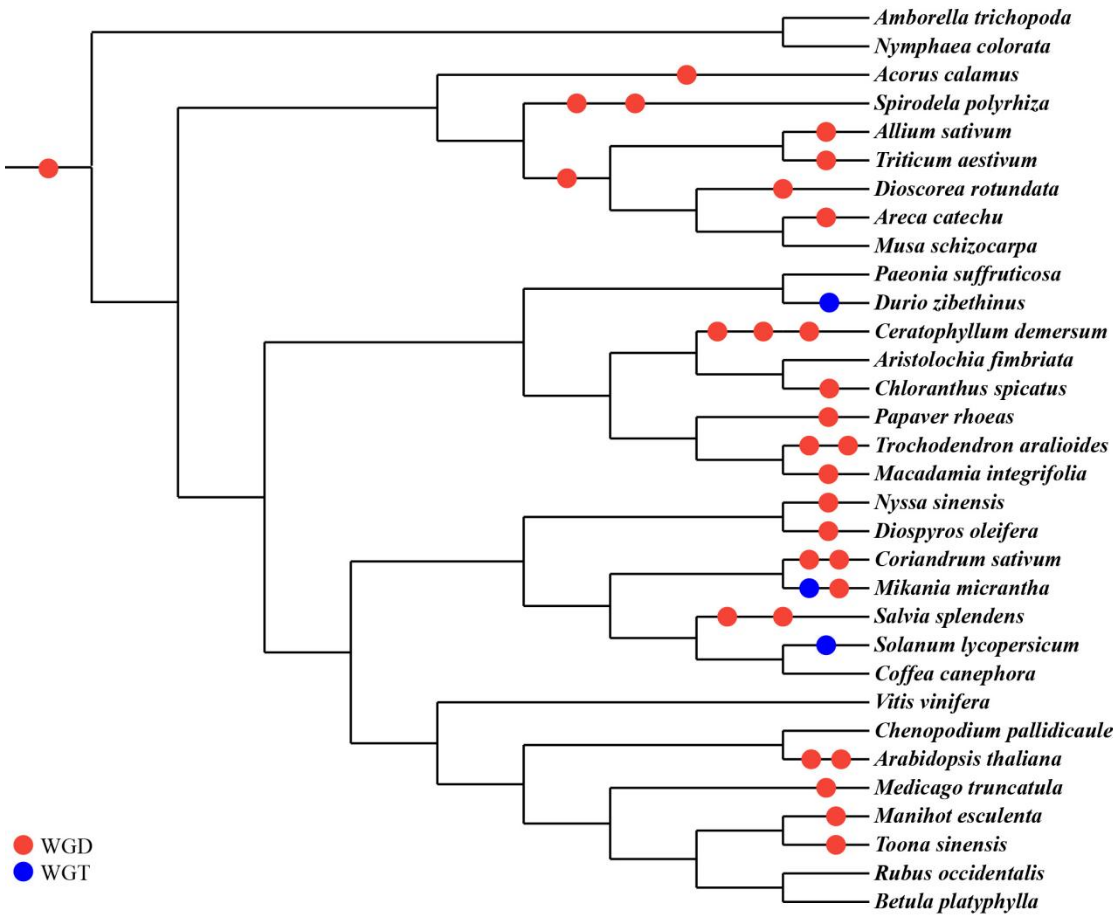

Fig S1. **The WGD events of Flowering species in class level.**

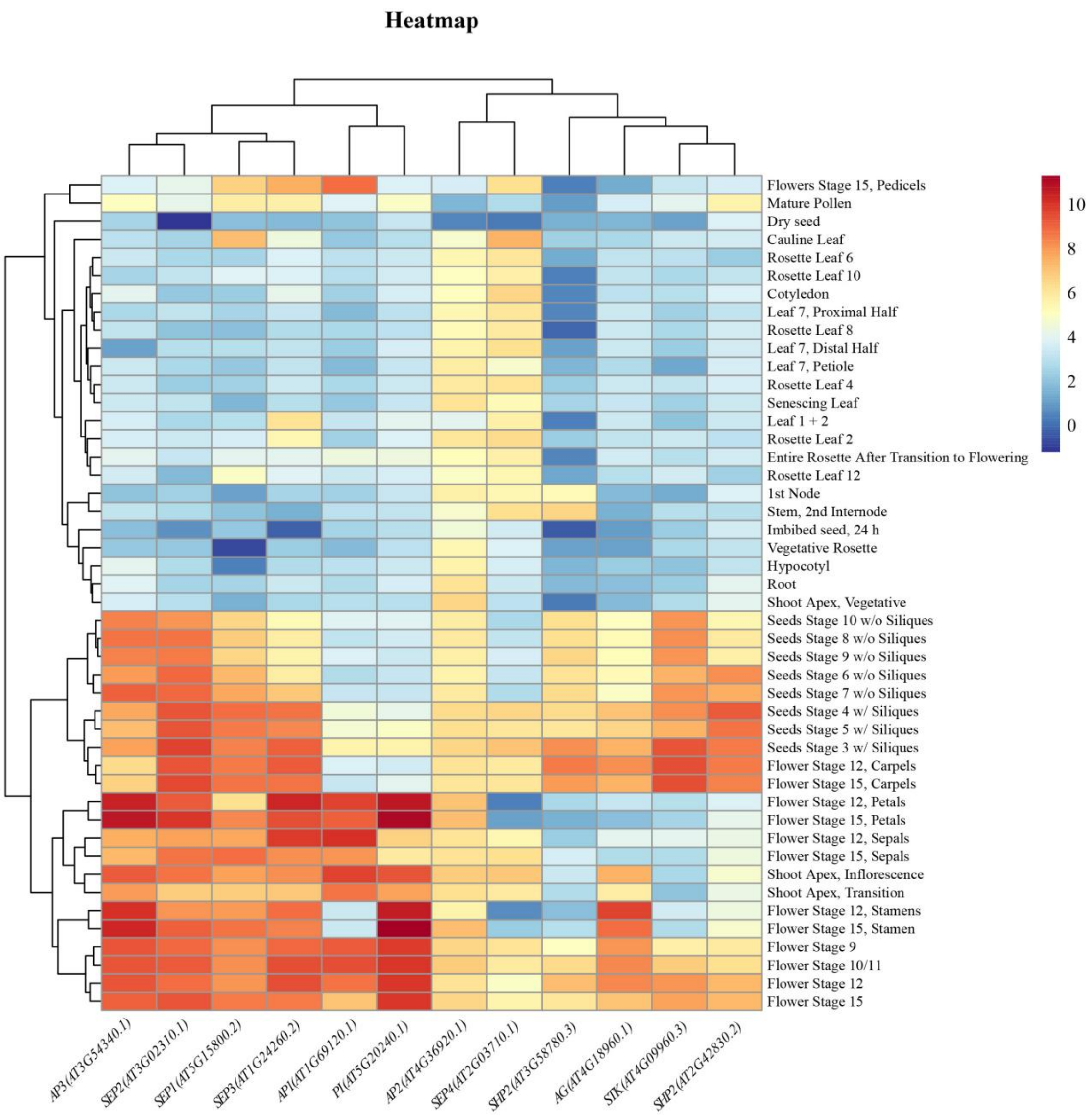

Fig S2. **The heatmap of ABCDE gene transcriptome data in *A. thaliana*.**

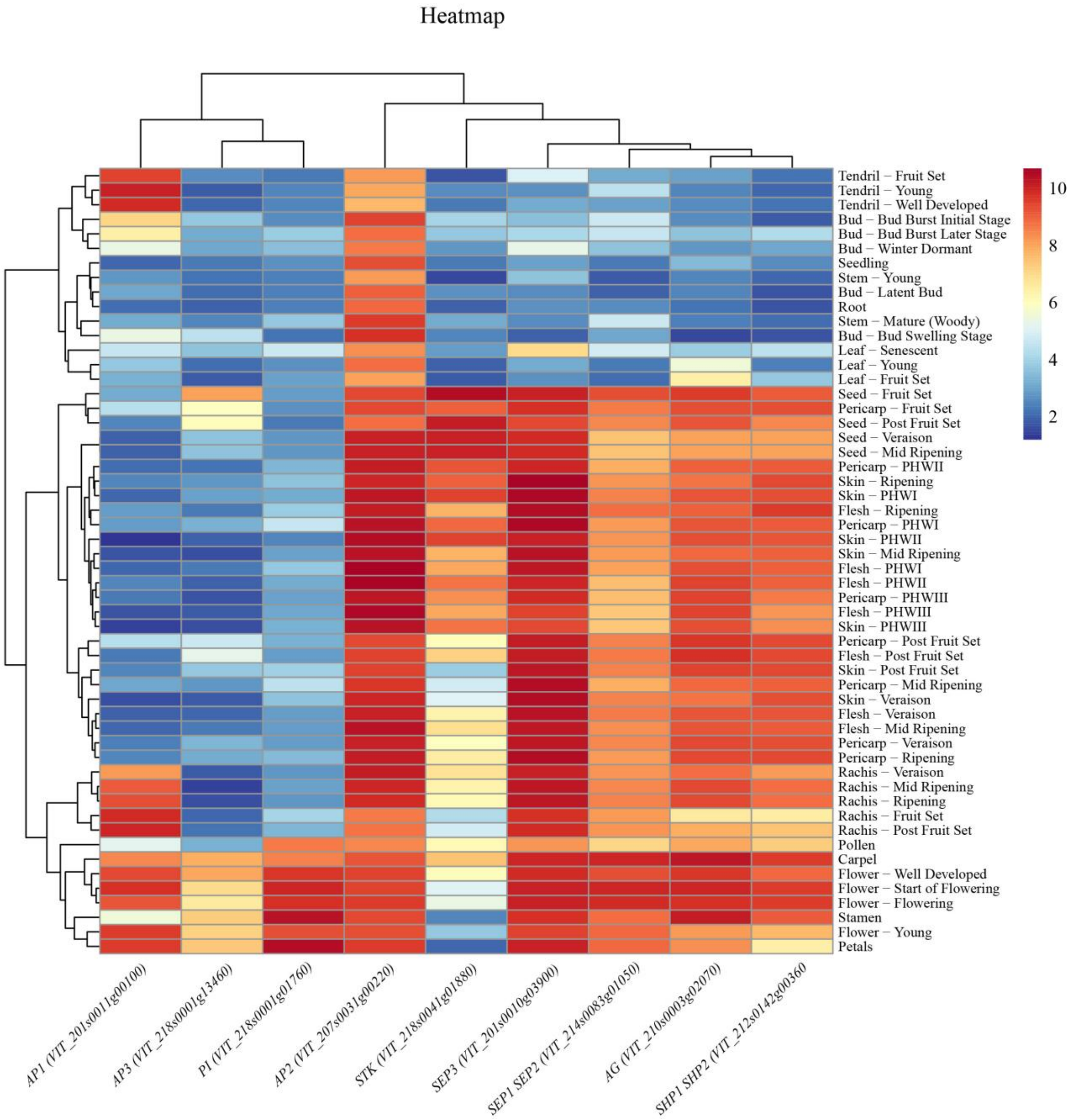

Fig S3. **The heatmap of ABCDE gene transcriptome data in *V. vinifera*.**

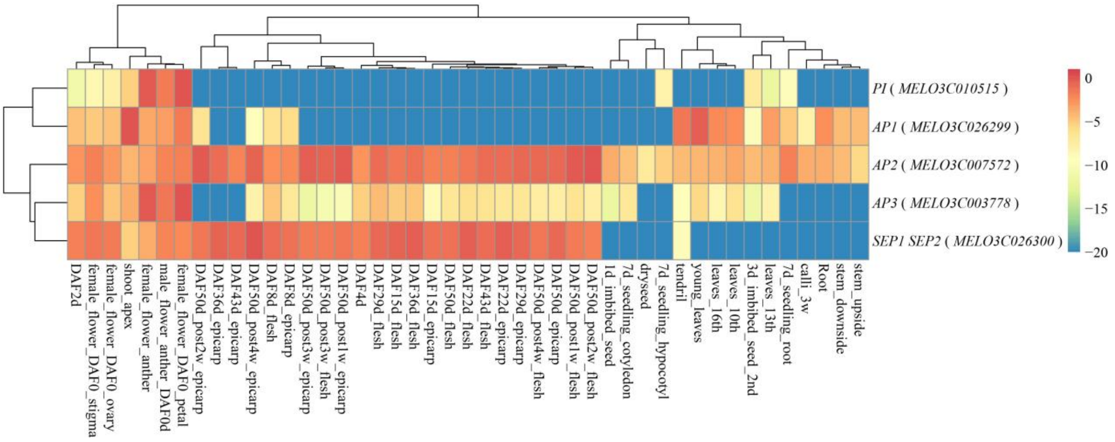

Fig S4. **The heatmap of ABCDE gene transcriptome data in *C. melo.***

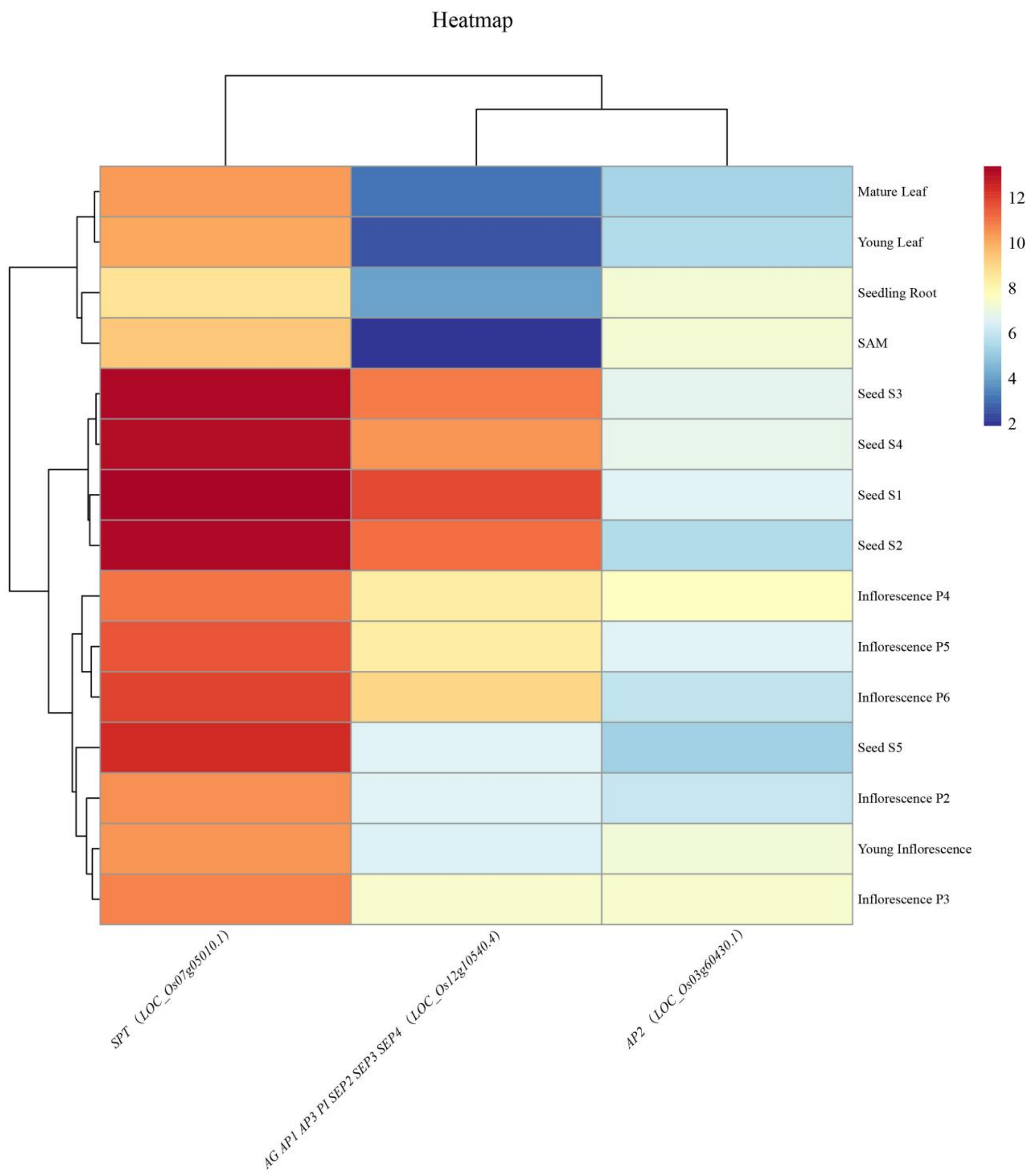

Fig S5. The heatmap of ABCDE gene transcriptome data in *O. sativa.*

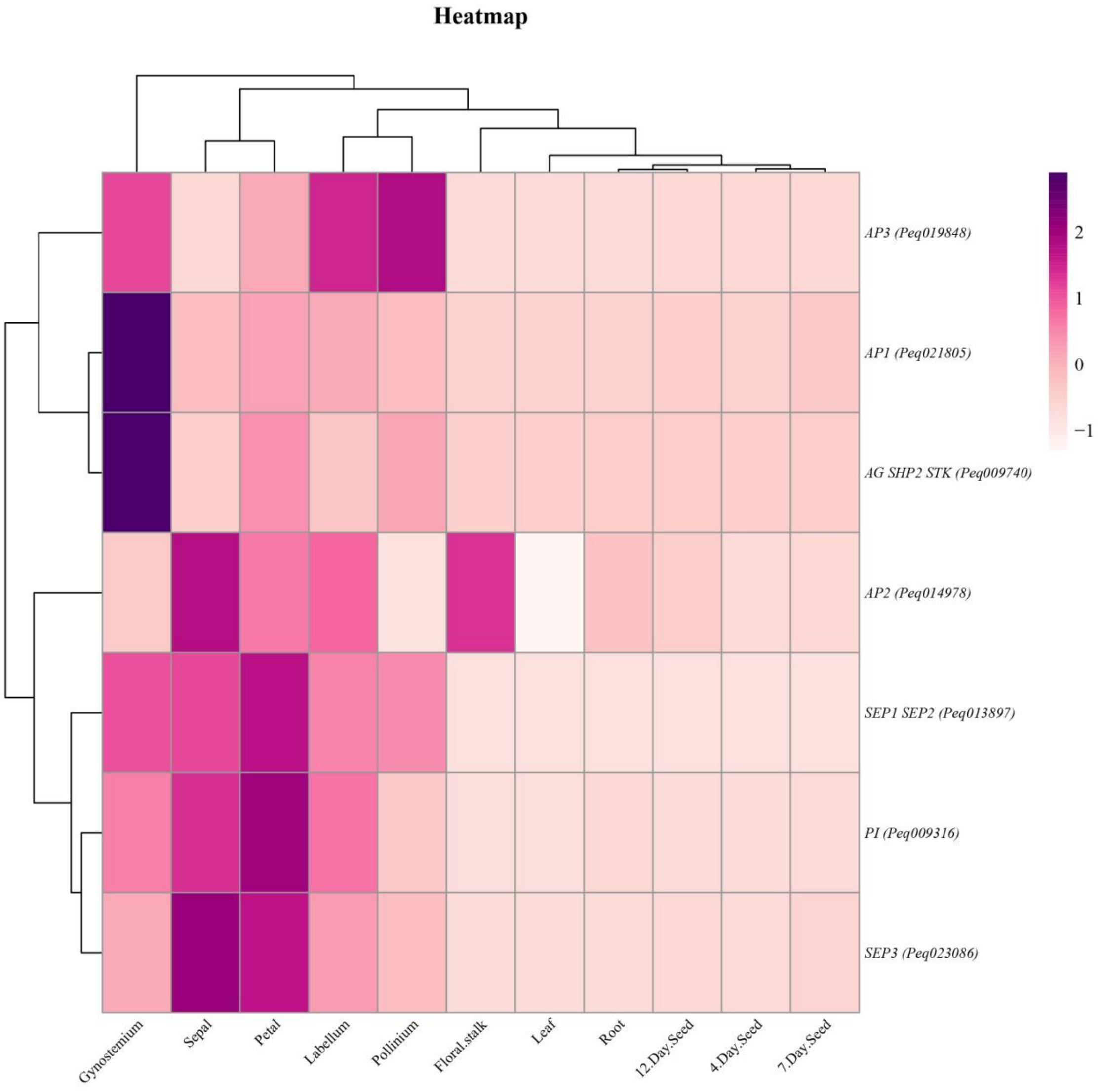

Fig S6. **The heatmap of ABCDE gene transcriptome data in *P. equestris.***

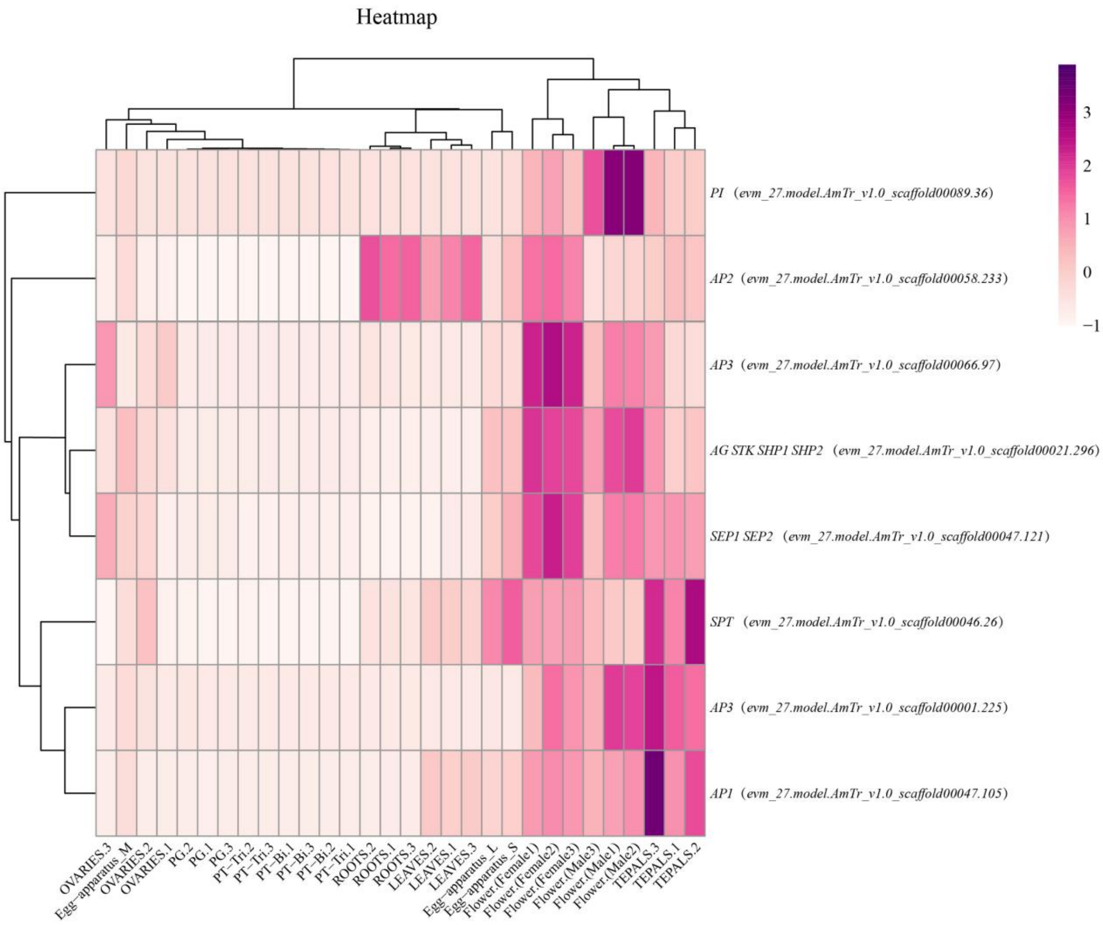

Fig S7. The heatmap of ABCDE gene transcriptome data in *A. trichopoda.*
